# Influence of respiratory disease experiences on COVID-19 vaccine acceptance: a study from Southeastern Louisiana

**DOI:** 10.3389/fpubh.2025.1593861

**Published:** 2025-08-15

**Authors:** Saba Barri, Sara Al-Dahir, Klaus Heyer, Ashley Taylor, Alaa Khalil, Mohamed Belkhouche, Brooke-Ashleigh Bonvillain, Kathryn Caldwell, Heather Surcouf, Ibrahim Hamed, Malack Jwayyed, Leslie S. Craig, Daniel F. Sarpong, Daniel Salmon

**Affiliations:** ^1^College of Pharmacy, Xavier University of Louisiana, New Orleans, LA, United States; ^2^Elaine P Nunez College, Chalmette, LA, United States; ^3^START Corporation, Covington, LA, United States; ^4^C & S Family Pharmacy, Metairie, LA, United States; ^5^Methodist Clinic, St. Bernard, LA, United States; ^6^Independent Researcher, Bridgetown, Barbados; ^7^Yale School of Medicine, Yale University, New Haven, CT, United States; ^8^Department of International Health, Bloomberg School of Public Health, Johns Hopkins University, Baltimore, MD, United States

**Keywords:** respiratory diseases, COVID-19, vaccination, vaccine behavior, public health interventions, attitudes

## Abstract

**Introduction:**

Respiratory diseases, including influenza (flu) and respiratory syncytial virus, continue to be major health concerns globally. The onset of COVID-19 further compounded these issues, making it important to examine public attitudes toward vaccination and understanding of respiratory diseases. This study explores factors influencing decisions to receive the latest COVID-19 vaccine, focusing specifically on the role of prior respiratory illness diagnosis.

**Methods:**

A follow-up survey among 299 participants from Southeastern Louisiana across 10 healthcare facilities was administered via phone and the Qualtrics platform, gathering information about the likelihood of receiving the latest COVID-19 vaccine. Quantitative data were analyzed using log-binomial and Poisson regression models to assess relationships between respiratory illness history and COVID-19 vaccine acceptance.

**Results:**

Nearly half (47%) of the participants reported a history of respiratory illness. Individuals with prior respiratory diagnosis were more likely to accept the latest COVID-19 vaccine (62%) than those without (41%) (RR: 1.79, 95% CI: 1.26–2.56). In fully adjusted models, accounting for age, prior vaccine hesitancy, and comorbidities, influenza vaccine acceptance (RR: 1.87, 95% CI: 1.06–3.28) was associated with greater likelihood of receiving the latest COVID-19 vaccine. Key barriers to vaccination, including concerns about side effects and distrust in vaccine efficacy were identified.

**Discussion:**

Participants with respiratory illnesses and those with positive vaccination histories, particularly regarding influenza, showed a higher likelihood of accepting the latest COVID-19 vaccines. However, significant obstacles to vaccine uptake persist. Tailored public health efforts that address these concerns are crucial to improving vaccine rates, particularly among hesitant groups.

## Introduction

Respiratory diseases, such as asthma, chronic obstructive pulmonary disease (COPD), influenza, and respiratory syncytial virus (RSV), have long posed significant challenges for global public health, accounting for millions of deaths each year (1, 2). The COVID-19 pandemic added complexity, overwhelming healthcare systems already burdened by respiratory conditions (3). As the pandemic ended, understanding public perceptions of these respiratory diseases in conjunction with COVID-19 vaccination efforts became critical for developing future public health strategies.

Co-infections, where a patient is simultaneously infected with more than one pathogen, have been a persistent issue in the management of respiratory diseases. Historically, co-infections have been linked to worsened clinical outcomes and increased complications in treatment (4, 5). This issue gained even more importance during the COVID-19 pandemic, where patients infected with both SARS-CoV-2 and other respiratory pathogens presented significant diagnostic and treatment challenges (2). Despite these complexities, there has been minimal focus on how public awareness of these co-infections might influence behavior, particularly regarding vaccine uptake.

Several studies have highlighted the impact of co-infections on respiratory disease management during the pandemic. In Ethiopia, researchers frequently detected SARS-CoV-2 co-infections with influenza and RSV, further complicating the treatment of respiratory illnesses (6). Similarly, non-pharmacological interventions (NPIs) such as mask-wearing and social distancing were effective in reducing the spread of certain viruses, but others, like rhinovirus, continued to circulate among children (7). In Arkansas, NPIs also significantly reduced pediatric infections caused by *Mycoplasma pneumoniae* and other respiratory viruses during the pandemic (8). The effectiveness of NPIs in reducing respiratory viruses such as RSV and influenza, particularly in children, raised critical questions about their role, compared to vaccines, in controlling outbreaks (9, 10).

While efforts such as NPIs and vaccination campaigns have been implemented to lessen the spread of respiratory viruses (11), significant gaps remain in understanding how public perception of respiratory diseases impacts vaccine behavior. Specifically, while research has largely focused on clinical outcomes of co-infections, there is limited exploration of how public awareness of respiratory diseases might influence health behaviors, including vaccine uptake (12, 13). This gap in research limits the ability of healthcare systems to fully address the complexities of managing multiple respiratory pathogens during future outbreaks.

In the United States, including Louisiana, individuals with chronic respiratory diseases such as asthma and COPD are strongly recommended to receive routine vaccinations, including the annual influenza and pneumococcal vaccines (e.g., PCV20 or a sequential schedule of PCV15 followed by PPSV23). These vaccines are crucial in reducing the risk of severe illness and complications. However, national and regional data show variable uptake among these groups, often influenced by individual risk perception and access to care (14, 15). This study aims to explore how individuals with respiratory diseases perceive the COVID-19 vaccine and how these perceptions influence their vaccination decisions. Given the ongoing spread of respiratory pathogens, understanding how public perception affects vaccination decisions could be crucial for enhancing future public health responses. The findings will provide insights into how public awareness of respiratory conditions influences vaccine behavior, ultimately contributing to more effective public health strategies during respiratory disease outbreaks.

## Materials and methods

### Study design and participants

This study employed a community based participatory research approach by engaging community healthcare partners from Southeastern Louisiana. This report was the final follow-up in a longitudinal survey which assessed participants’ likelihood of keeping up to date with the COVID-19 vaccine series, i.e., receiving the latest COVID-19 vaccine, with a focus on how perceptions of respiratory illness influenced decision-making. Initially, the baseline survey identified varying levels of vaccine acceptance, influenced by demographic factors and previous health experiences, laying the groundwork for understanding shifts in attitudes over time (16). A total of 299 participants, who were part of the baseline survey, were included. A total of 79.3% of recipients were retained from baseline, with over 90% of follow-up responses occurring via in-person follow-up. Participants were recruited from 10 healthcare locations across Southeastern Louisiana, including pharmacies and clinics serving rural and marginalized communities. Data collection for this phase focused on participants’ likelihood of getting the latest COVID-19 vaccine and their perspectives on respiratory diseases.

### Data collection

Trained pharmacists and healthcare workers conducted data collection in-person or through phone interviews, with surveys administered via the Qualtrics© platform (17). All baseline surveys were conducted in Summer and Fall 2022. All follow-up surveys were conducted within 12-months, from August through November 2023. The survey gathered self-reported quantitative data regarding participants’ likelihood of receiving the latest COVID-19 vaccine, along with information on respiratory illness diagnoses and other health-related concerns. All vaccine status information was validated by the healthcare worker via their health informatics system. The survey questions focused on identifying factors that influenced participants’ decision-making regarding vaccination and their general attitudes toward respiratory health.

### Variable definitions

Surveys were designed to capture demographic characteristics, respiratory diagnosis history, chronic disease history, COVID-19 experiences and perceptions, including vaccination hesitancy, and other behavioral measures, via self-report. Demographic characteristics included age (17–49 years vs. 50 years and above), race (categorized as African American, Caucasian, or Other), gender (male vs. female), and area of residence (rural/semi-rural vs. suburban/city). Respiratory illness history included conditions such as flu, cold, allergy, sinus infection, ear infection, pneumonia, throat infection, RSV, strep throat, or other respiratory diseases except COVID-19 diagnosed between Fall 2022 and Fall 2023. Respiratory illness, whether physician diagnosed or self limiting requiring only over-the counter management, were included as one predictive variable without differentiation of severity of illness. This was done to ensure capturing all clinically relevant respiratory infections. Presence of chronic health conditions, such as diabetes, hypertension, or cardiovascular diseases, were also recorded. COVID-19 diagnosis captured whether participants had tested positive for COVID-19 during the study period. Post-vaccination COVID-19 infection assessed whether participants tested positive for COVID-19 after receiving a COVID-19 vaccine. The latest COVID-19 vaccine reflected the vaccination released within the cohort year of the participation. This study spanned vaccination releases in both 2022 and 2023. Latest vaccination was referenced according to the most current release at the time of the survey administration. Vaccination status was confirmed via pharmacy and clinic records, as well as patient self-report. The vaccine hesitancy score was calculated based on participants’ responses to questions addressing concerns about vaccine safety, efficacy, and the potential for side effects. Scores were categorized into tertiles: not hesitant, moderately hesitant, and most hesitant. Behavioral measures included previous refusal of any vaccines, likelihood of receiving an influenza vaccine and willingness to get vaccinated during a new pandemic or rapidly spreading infection. Participants’ opinions about COVID-19 as a public health risk were also evaluated. All survey instruments were drawn from previously validated tools in the research groups COVID-19 studies and are described in referenced publications (16).

### Data analysis

The data were analyzed using STATA version 18 (18). Descriptive statistics such as frequencies and proportions were calculated to summarize demographic characteristics, responses about vaccine acceptance, and respiratory illness history. The primary outcome variable was the participants’ likelihood of receiving the latest COVID-19 vaccine. A log-binomial regression model was used to explore associations between respiratory illness diagnoses and the likelihood of vaccine uptake. In cases where model convergence issues occurred, Poisson regression was employed. The final model was adjusted for variables that might confound the relationship between respiratory illness and vaccine uptake. These adjustments included age, gender, race/ethnicity (African American, Caucasian, Others), vaccine hesitancy (not hesitant, moderately hesitant, most hesitant), prior vaccine refusal, history of COVID-19 diagnosis, perceptions of COVID-19 as a public health risk, and other health conditions. Such adjustments were critical to ensure the accuracy of our findings by mitigating potential biases and providing a clearer picture of the factors influencing vaccine uptake.

## Results

A total of 299 participants were included in this final follow-up survey. Among them, 47% (141) reported a history of respiratory disease. The data in Table 1 show that there was a significant association between a previous COVID-19 diagnosis and a history of respiratory disease (RR: 1.49, 95% CI: 1.15–1.91, *p* = 0.002), where participants with a history of respiratory illness were more likely to have been diagnosed with COVID-19 during the study period. Participants diagnosed with respiratory illness were significantly more likely to report having chronic health conditions compared to those not diagnosed (67% vs. 46%; RR: 1.58, 95% CI: 1.21–2.06, *p* = 0.001). Additionally, significant differences were observed in participants’ likelihood to receive vaccinations, with a higher proportion of those diagnosed with respiratory illness indicating willingness to get the vaccine during a new pandemic or rapidly spreading infection (61% vs. 36%, RR: 1.71, 95% CI: 1.33–2.19, *p* < 0.001) and also indicating a higher likelihood of receiving the influenza vaccine (47% vs. 31%; RR: 1.41, 95% CI: 1.11–1.78, *p* = 0.004).

**Table 1:** Baseline characteristics of participants based upon exposure to respiratory disease diagnosis in Fall 2022 through Fall 2023.

Covariates	Not diagnosed with	Diagnosed with	RR (95%CI) (*p*-value)
	respiratory disease	respiratory disease	
	(*N* = 158)	(*N* = 141)	
	Frequency and percentage		
Age			
17–49 (ref)	114 (72%)	104 (74%)	—
50 and above	44 (28%)	37 (26%)	0.958 (0.727, 1.26) *0.757*
Race			
African American (ref)	62 (39%)	49 (35%)	—
Caucasian	80 (51%)	70 (50%)	1.06 (0.807, 1.39) *0.687*
Other	16 (10%)	22 (16%)	1.31 (0.931, 1.85) *0.121*
Gender			
Male (ref)	53 (34%)	48 (34%)	—
Female	105 (66%)	93 (66%)	0.989 (0.768, 1.27) *0.927*
Area of residence			
Rural/Semirural (ref)	42 (27%)	43 (31%)	—
Suburb/City	116 (73%)	98 (69%)	0.905 (0.701, 1.17) *0.445*
Have you ever refused a vaccine?			
No (ref)	96 (61%)	84 (60%)	—
Yes	62 (39%)	57 (40%)	1.03 (0.804, 1.31) *0.834*
Vaccine hesitancy score (tertiles)			
Not hesitant (ref)	88 (56%)	87 (62%)	—
Moderately hesitant	28 (18%)	20 (14%)	0.838 (0.581, 1.21) *0.345*
Most hesitant	42 (27%)	34 (24%)	0.900 (0.673, 1.20) *0.477*
Have you been diagnosed with COVID-19? (Between September 2022 and today)			
No (ref)	142 (90%)	111 (79%)	—
Yes	16 (10%)	30 (21%)	1.49 (1.15, 1.91) ***0.002***
If there is another major pandemic or rapidly spreading NEW infection, how likely are you to get the vaccine if it is a new vaccine for a new virus?			
Not likely (ref)	101 (64%)	55 (39%)	—
Likely	57 (36%)	86 (61%)	1.71 (1.33, 2.19) < ***0.001***
Did you get the COVID infection after getting the vaccine?			
No (ref)	129 (82%)	104 (74%)	—
Yes	29 (18%)	37 (26%)	1.26 (0.97, 1.62) *0.082*
How likely are you to receive the flu vaccines for Fall 2023?			
Not likely (ref)	109 (69%)	75 (53%)	—
Likely	49 (31%)	66 (47%)	1.41 (1.11, 1.78) ***0.004***
Do you have any health conditions?			
No (ref)	85 (54%)	47 (33%)	—
Yes	73 (46%)	94 (67%)	1.58 (1.21, 2.06) ***0.001***
What is your current opinion about COVID-19 disease?			
COVID-19 is not a public health risk (ref)	80 (51%)	66 (47%)	
COVID-19 is still a public health risk	78 (49%)	75 (53%)	1.08 (0.852, 1.38) 0.510

RR, risk ratio; 95% CI, confidence interval. Italicized values indicates nonsignificant results; bolded values indicate statistically significant results (*p* < 0.05).

Table 2 shows that for participants aged 50 years and above, a significantly greater proportion (37% compared to 23%) were likely to get the latest COVID-19 vaccine compared to those aged under 50 years (RR: 1.61, 95% CI: 1.14–2.25, *p* = 0.006). Caucasians were less likely to accept the latest COVID-19 vaccine than African Americans (RR: 0.652, 95% CI: 0.451–0.941, *p* = 0.023). Participants with a history of refusing previous vaccines were also significantly less likely to accept the latest COVID-19 vaccine (RR: 0.672, 95% CI: 0.460–0.983, *p* = 0.041). Participants with moderate and high hesitancy scores showed significant associations with a lower likelihood of accepting the latest COVID-19 vaccine. Moderate hesitancy was associated with a reduced likelihood of vaccine acceptance (RR: 0.393, 95% CI: 0.193–0.800, *p* = 0.010), while high hesitancy further decreased the likelihood (RR: 0.213, 95% CI: 0.096–0.469, *p* < 0.001). Conversely, participants with a history of respiratory disease were more likely to accept the latest COVID-19 vaccine (RR: 1.79, 95% CI: 1.26–2.56, *p* = 0.001). Participants who were likely to get the influenza vaccine were also more likely to accept the latest COVID-19 vaccine (RR: 4.47, 95% CI: 2.98–6.69, *p* < 0.001).

**Table 2:** Predictors of likely to get latest COVID-19 vaccine among the study participants.

Covariates	Not likely to get the latest COVID-19 vaccine	Likely to get the latest COVID-19 vaccine	RR (95%CI) (*p*-value)
	(*N* = 208)	(*N* = 91)	
	Frequency and percentage		
Age			
17–49 (ref)	161 (77%)	57 (63%)	—
50 and above	47 (23%)	34 (37%)	1.61 (1.14, 2.25) ***0.006***
Race			
African American (ref)	69 (33%)	42 (46%)	—
Caucasian	113 (54%)	37 (41%)	0.652 (0.451, 0.941) ***0.023***
Other	26 (12%)	12 (13%)	0.974 (0.494, 1.41) *0.500*
Gender			
Male (ref)	71 (34%)	30 (33%)	—
Female	137 (66%)	61 (67%)	1.04 (0.720, 1.49) *0.845*
Area of residence			
Rural/Semirural (ref)	61 (29%)	24 (26%)	—
Suburb/City	147 (71%)	67 (74%)	1.11 (0.749, 1.64) *0.606*
Have you ever refused a vaccine?			
No (ref)	117 (56%)	63 (69%)	—
Yes	91 (44%)	28 (31%)	O.672 (0.460, 0.983) ***0.041***
Vaccine hesitancy score (tertiles)			
Not hesitant (ref)	110 (50%)	65 (83%)	—
Moderately hesitant	41 (19%)	7 (10%)	0.393 (0.193, 0.800) ***0.010***
Most hesitant	70 (32%)	6 (8%)	0.213 (0.096, 0.469) < ***0.001***
Have you been diagnosed with COVID-19? (Between September 2022 and today)			
No (ref)	181 (87%)	72 (79%)	—
Yes	27 (13%)	19 (21%)	1.45 (0.977, 2.16) *0.065*
If there is another major pandemic or rapidly spreading NEW infection, how likely are you to get the vaccine if it is a new vaccine for a new virus?			
Not likely (ref)	183 (88%)	21 (23%)	—
Likely	25 (12%)	70 (77%)	7.16 (4.69, 10.9) < ***0.001***
Did you get the COVID infection after receiving the COVID-19 vaccine?			
No (ref)	174 (84%)	59 (65%)	—
Yes	34 (16%)	32 (35%)	1.91 (1.37, 2.67) < ***0.001***
How likely are you to receive the flu vaccines?			
Not likely (ref)	160 (77%)	24 (26%)	—
Likely	48 (23%)	67 (74%)	4.47 (2.98, 6.69) < ***0.001***
Do you have any chronic health conditions?			
No (ref)	103 (50%)	29 (31%)	—
Yes	105 (50%)	62 (68%)	1.69 (1.16, 2.46) ***0.006***
What is your current opinion about COVID-19 disease?			
COVID-19 is not a public health risk (ref)	112 (54%)	34 (37%)	
COVID-19 is still a public health risk	96 (46%)	57 (63%)	1.60 (1.12, 2.29) **0.010**
Have you been diagnosed with any respiratory illness in Fall 2022 through Fall 2023?			
No (ref)	123 (59%)	35 (38%)	—
Yes	85 (41%)	56 (62%)	1.79 (1.26, 2.56) ***0.001***

Italicized values indicates nonsignificant results; bolded values indicate statistically significant results (*p* < 0.05).

**Figure 1:**
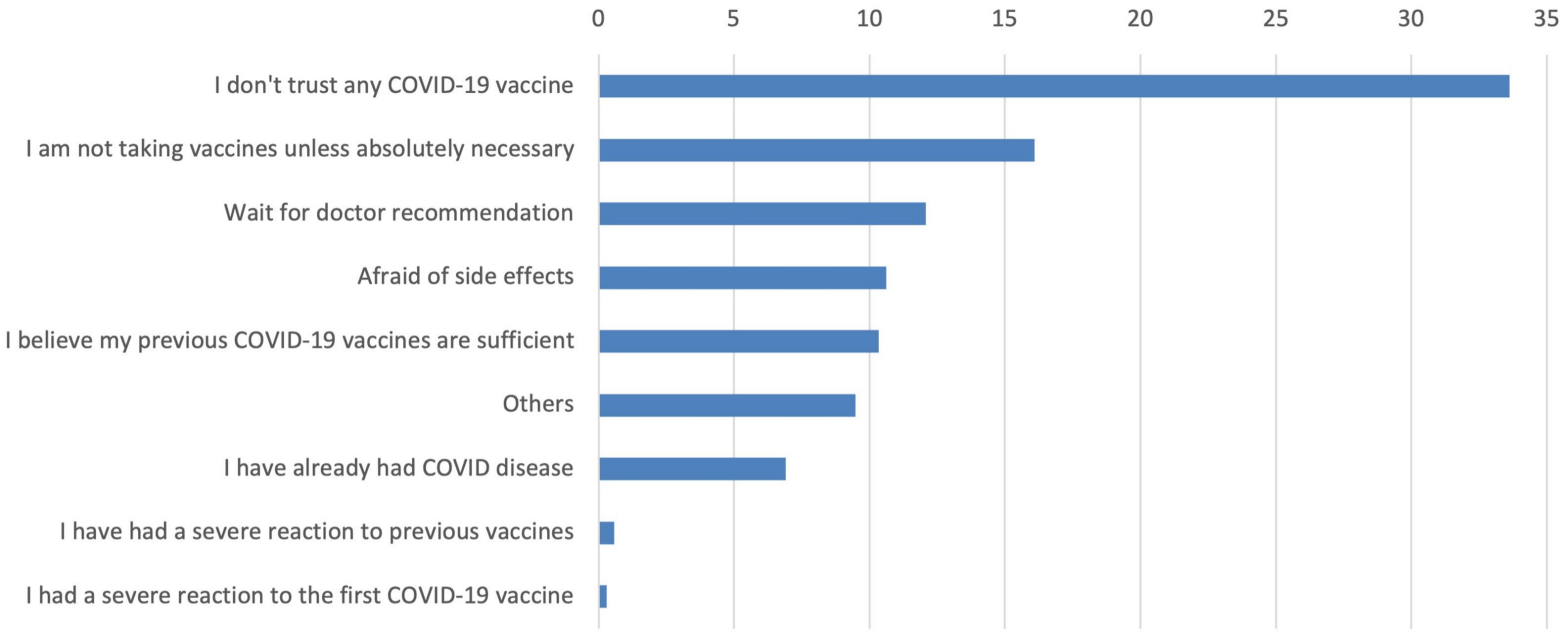
The reason the people are not interested in latest COVID-19 vaccine.

Figure 1 shows the reasons why participants indicated they were not interested in receiving the latest COVID-19 vaccine. The most frequently cited reason was not trusting any COVID-19 vaccine (33.6%), followed by views about only taking vaccines if absolutely necessary (16.1%), waiting for a doctor’s recommendation (12.1%) and concerns about side effects (10.6%).

The final model variables displayed in Table 3 illustrate the likelihood of accepting the latest COVID-19 vaccine. The unadjusted model showed a significant association between respiratory illness and COVID-19 vaccine acceptance (RR: 1.79, 95% CI: 1.18–2.74, *p* = 0.007). While age, gender, and chronic health conditions were not statistically significant in the fully adjusted model, participants likely to get the influenza vaccine showed a significantly higher likelihood of accepting the COVID-19 vaccine (RR: 1.87, 95% CI: 1.06–3.28, *p* = 0.030), compared to those were unlikely. Additionally, those willing to vaccinate during a new pandemic exhibited the strongest association (RR: 5.09, 95% CI: 2.76–9.40, *p* < 0.001).

**Table 3:** Final model variable for likelihood to getting updated COVID-19 vaccine.

	RR	95% CI, *p-value*
Unadjusted (Participant diagnosed with respiratory illness but not COVD-19 since Fall 2022)	1.79	(1.18, 2.74) *0.007*
Variable		
Participant diagnosed with respiratory illness but not COVD-19 since Fall 2022 (yes)	1.09	(0.689, 1.74) *0.702*
Age greater than 50 years old	0.748	(0.463, 1.21) *0.237*
Race		
African American (ref)	–	–
Caucasian	0.732	(0.454, 1.18) *0.201*
Others	0.933	(0.467, 1.86) *0.844*
Gender (Male)	0.818	(0.508, 1.32) *0.408*
Vaccine hesitancy score (tertiles)		
Not hesitant (ref)	–	–
Moderately hesitant	0.898	(0.431, 1.87) *0.774*
Most hesitant	0.792	(0.391, 1.61) *0.518*
Have you ever refused a vaccine (Yes)	1.13	(0.697, 1.82) *0.627*
COVID-19 diagnosis (Yes)	1.28	(0.729, 2.23) *0.393*
How likely are you to get the vaccine for another major pandemic or rapidly spreading old infection (Likely)	5.09	(2.76, 9.40) < ***0.001***
Covid diagnosis after getting the vaccine (Yes)	1.17	(0.726, 1.90) *0.513*
How likely are you to get the flu vaccine (Likely)	1.87	(1.06, 3.28) ***0.030***
Do you have any health conditions (Yes)	1.43	(0.881, 2.32) *0.148*
What is your current opinion about COVID-19 disease? (yes)	0.809	(0.510, 1.28) *0.368*

RR, risk ratio; 95% CI, confidence interval. Italicized values indicates nonsignificant results; bolded values indicate statistically significant results (*p* < 0.05).

## Discussion

Individuals with a history of respiratory illness were significantly more willing to receive the latest COVID-19 vaccine, in this sample, highlighting the role of perceived vulnerability in shaping vaccine behavior. Research consistently shows that individuals with chronic respiratory conditions prioritize preventive health measures, including vaccination, to lessen their risk of severe health outcomes (19–21). In Southeastern Louisiana, this relationship may be further amplified by the region’s high prevalence of respiratory illnesses and limited healthcare resources, creating a sense of urgency among vulnerable populations (22). This is particularly relevant in the context of COVID-19, where awareness of potential complications likely drives health-protective behaviors (23). Additionally, these findings align with health behavior theories, which emphasize that perceptions of personal risk are critical motivators for engaging in preventive actions, such as vaccination (24). Participants with respiratory disease likely have increased interaction with healthcare providers due to their underlying conditions, increasing their opportunities for vaccine education and trust-building during routine care (23, 25). This suggests that healthcare engagement plays an important role in enhancing vaccine acceptance, particularly for populations at higher risk. Although our study did not assess vaccination opportunities during hospitalization, prior research highlights the potential impact of leveraging inpatient and outpatient care settings to offer recommended vaccines. Studies have shown that routine vaccination during hospitalization for chronic patients, especially those with respiratory illnesses, can significantly increase coverage and reduce missed opportunities (15, 26). This suggests that future interventions might benefit from integrating vaccine delivery into standard chronic disease management visits. The alignment of these findings with the behavioral theories, such as the Health Belief Model, reinforces the importance of utilizing healthcare interactions to address hesitancy and promote vaccine uptake among high-risk individuals (27, 28).

Influenza vaccine acceptance emerged as a strong predictor in latest COVID-19 vaccine uptake as well, suggesting that integrating multiple vaccine campaigns, for example, combining influenza vaccine campaigns with COVID-19 vaccine outreach, could enhance overall vaccine uptake (20, 29). Although our study did not examine booster uptake or co-administration directly, recent research supports the safety and feasibility of administering multiple vaccines simultaneously in high-risk populations, including individuals with chronic respiratory conditions. These findings highlight the value of integrated vaccine delivery models, particularly in outpatient settings, where vaccine recommendations can be reinforced during routine care (30–32). Willingness to vaccinate during a new pandemic or rapidly spreading infection was another strong predictor of COVID-19 vaccine uptake (RR: 5.09, 95% CI: 2.76–9.40, *p* < 0.001), emphasizing the influence of perceived vulnerability. These results highlight the complex and multifactorial nature of vaccine uptake, where factors such as prior positive vaccine behaviors, perceptions of risk, and individual trust in vaccines converge. For instance, moderate hesitancy reduced the likelihood of vaccine acceptance (RR: 0.798, 95% CI: 0.567–0.912, *p* = 0.021), while high hesitancy further decreased this likelihood (RR: 0.612, 95% CI: 0.433–0.873, *p* = 0.007).

Mistrust in vaccines emerged as a significant barrier, with 33.62% of participants citing distrust in COVID-19 vaccines as the primary reason for not accepting, followed by preferences for vaccines if it was necessary (16.09%), waiting for a doctor’s recommendation (12.07%), and concerns about side effects (10.63%). These findings underscore the deeply rooted mistrust in vaccines and highlight critical areas for targeted public health interventions to address hesitancy and misinformation. By focusing on these barriers and promoting trust in healthcare providers, public health strategies can better address the interplay of factors influencing vaccine uptake (23). Beyond addressing individual concerns, broader organizational and communication strategies are critical in reducing vaccine hesitancy. Evidence suggests that well-structured health communication campaigns, combined with the trusted role of healthcare workers (HCWs), can significantly improve vaccine confidence and uptake. HCWs serve as influential messengers, especially when interventions are personalized and culturally appropriate (33, 34). Incorporating educational outreach and clear communication strategies into routine care may help overcome barriers related to misinformation, mistrust, and limited health literacy.

Vaccine hesitancy persists as a critical barrier, specifically among underrepresented communities in Southeastern Louisiana. Concerns about vaccine side effects, mistrust in efficacy, and logistical challenges reflect longstanding issues of healthcare inequity (35, 36). These barriers align with other studies on vaccine hesitancy, highlight the impact of misinformation and logistical challenges on vaccine decisions (27, 36). Historical healthcare discriminations, particularly affecting African American communities, have exacerbated these challenges and underline the need for culturally tailored public health interventions (35, 37). In Southeastern Louisiana, addressing these issues is important given the region’s unique demographic and health disparities, that may amplify these challenges among marginalized communities (22). Public health campaigns should focus on educating individuals about vaccine safety and efficacy, addressing common fears and misconceptions that lead to hesitancy. Moreover, logistical barriers such as access to vaccination sites should be considered in future outreach efforts to ensure equitable access to vaccines.

## Strengths and limitations

This study has several limitations that should be in consideration. First, the study did measure trust in healthcare providers and institutions; yet, these were not found to be significant in the bivariate or multivariate analysis and excluded from model prediction. Healthcare trust is a factor consistently highlighted in previous research as a critical determinant of vaccine acceptance, particularly in Louisiana (38, 39). Additionally, the study did not include an analysis of preventive health behaviors beyond influenza vaccine acceptance, which could have provided further insights into vaccine decision-making patterns. Addressing these gaps in future research would enhance the understanding of the multifactorial drivers of vaccine acceptance.

To address selection bias, participants were randomly sampled from various healthcare locations across Southeastern Louisiana, which aimed to minimize bias and enhance the representativeness of the sample. However, as with any study, the potential for selection bias cannot be completely ruled out. The random sampling method was intended to distribute any unmeasured confounders evenly among the study groups, thus reducing their impact on the observed associations. Future studies could improve on this by incorporating stratified or cluster sampling techniques, especially when targeting specific sub populations or regions.

Despite these limitations, the study offers significant strengths. The focus on actionable predictors, including respiratory disease history, vaccine hesitancy, and influenza vaccine behavior, allows for a detailed exploration of factors that directly influence COVID-19 vaccine uptake. Furthermore, the study’s diverse sample enhances its applicability to broader populations, providing critical insights for designing equitable public health strategies. These findings are consistent with prior research emphasizing the importance of targeted approaches to addressing disparities in vaccine acceptance (38, 39). By emphasizing predictors that are both practical and relevant, the findings contribute to a growing body of evidence supporting tailored interventions to address vaccine hesitancy and improve vaccination rates in underserved communities.

Table 3 underscores the complex relationship between predictors and vaccine acceptance. Specifically, this final model integrated key predictors such as respiratory disease history, vaccine hesitancy, and influenza vaccine behavior, providing a comprehensive perspective on the factors driving vaccine uptake. The inclusion of these variables emphasizes their theoretical and practical relevance in shaping public health strategies. This approach helps shape a clear and meaningful narrative, offering valuable insights for designing targeted interventions to address vaccine hesitancy and boost vaccine uptake in future public health efforts.

## Conclusion

This study provides valuable insights into the factors influencing the likelihood of receiving the latest COVID-19 vaccine, particularly highlighting the impact of respiratory disease history and influenza vaccine behavior. Participants with a history of respiratory illness were significantly more likely to express willingness to receive the latest COVID vaccine, reflecting their increased health concerns and likelihood of engaging in preventive health behaviors (19). This increased willingness demonstrates the critical role of personal risk perceptions in shaping health-protective behaviors. In Southeastern Louisiana, a region characterized by significant health disparities, these findings emphasize the need to consider regional contexts in vaccine strategies.

Overall, future public health strategies should encourage uptake of health-promoting behaviors, improve trust through transparent communication, and address logistical barriers to ensure broad and fair vaccine coverage (23, 36, 38). By focusing on both medical and social determinants of vaccine behavior, these strategies can strengthen preparedness for future pandemics or health crises (40). Understanding how the personal perceptions of respiratory disease influence vaccine behavior will be crucial for designing effective public health interventions in Southeastern Louisiana and beyond.

## Data Availability

The raw data supporting the conclusions of this article will be made available by the authors without undue reservation.
